# Feasibility, Acceptability, and Effectiveness of Enhanced Cognitive Behavioral Therapy (eCBT) for Children and Adolescents With Obsessive-Compulsive Disorder: Protocol for an Open Trial and Therapeutic Intervention

**DOI:** 10.2196/24057

**Published:** 2020-12-18

**Authors:** Lidewij H Wolters, Bernhard Weidle, Lucía Babiano-Espinosa, Norbert Skokauskas

**Affiliations:** 1 Department of Mental Health Regional Centre for Child and Youth Mental Health and Child Welfare (RKBU Central Norway) Norwegian University of Science and Technology (NTNU) Trondheim Norway; 2 de Bascule/Levvel Academic Center for Child and Adolescent Psychiatry Amsterdam Netherlands; 3 Department of Child and Adolescent Psychiatry St Olavs University Hospital Trondheim Norway

**Keywords:** obsessive-compulsive disorder, cognitive behavioral therapy, e-mental health, children, adolescents, cognitive, behavioral, pediatric

## Abstract

**Background:**

Although the evidence base of cognitive behavioral therapy (CBT) for pediatric obsessive-compulsive disorder (OCD) has been broadly established, the treatment is hampered by limited access, poor compliance, and nonresponse. New technologies offer the opportunity to improve the accessibility, user friendliness, and effectiveness of traditional office-based CBT. By employing an integrated and age-appropriate technologically enhanced treatment package, we aim to execute a more focused and attractive application of CBT principles to increase the treatment effect for pediatric OCD.

**Objective:**

The aim of this open study is to explore the acceptability, feasibility, and effectiveness of a newly developed enhanced CBT (eCBT) package for pediatric OCD.

**Methods:**

This study is an open trial using a historical control design conducted at the outpatient clinic of the Department of Child and Adolescent Psychiatry at St. Olavs University Hospital (Trondheim) or at BUP Klinikk (Aalesund). Participants are 30 children (age 7-17 years) with a primary Diagnostic and Statistical Manual of Mental Disorders (DSM)-5 diagnosis of OCD, and their parents. All participants receive eCBT. eCBT consists of the usual evidence-based CBT for pediatric OCD in an “enhanced” format. Enhancements include videoconferencing sessions (supervision and guided exposure exercises at home) in addition to face-to-face sessions; an app system of interconnected apps for the child, the parents, and the therapist; psychoeducative videos; and frequent online self-assessments with direct feedback to patients and the therapist. Primary outcome measures are the Children’s Yale-Brown Obsessive Compulsive Scale (CY-BOCS) (effectiveness), the Client Satisfaction Questionnaire-8 (acceptability), and treatment drop out (feasibility). Assessments are conducted pretreatment, posttreatment, and at 3- and 6-month follow-ups. A 12-month follow-up assessment is envisioned. The treatment outcome (CY-BOCS) will be compared to traditional face-to-face CBT (data collected in the Nordic Long-term OCD Treatment Study).

**Results:**

Ethical approval has been obtained (2016/716/REK nord). Inclusion started on September 04, 2017. Data collection is ongoing.

**Conclusions:**

This study is the first step in testing the acceptability, feasibility, and preliminary effectiveness of eCBT. In case of positive results, future steps include improving the eCBT treatment package based on feedback from service users, examining cost-effectiveness in a randomized controlled trial, and making the package available to clinicians and other service providers treating OCD in children and adolescents.

**Trial Registration:**

ISRCTN, ISRCTN37530113; registered on January 31, 2020 (retrospectively registered); https://www.isrctn.com/ISRCTN37530113.

**International Registered Report Identifier (IRRID):**

DERR1-10.2196/24057

## Introduction

### Background

Pediatric obsessive-compulsive disorder (OCD) is a relatively common, severe, and debilitating condition [1], leading to substantial impairment in family, academic, and social functioning [2,3] and reduced quality of life [4]. Cognitive behavioral therapy (CBT) is the treatment of choice [5,6], and its effectiveness has been extensively demonstrated [7-9]. However, treatment for pediatric OCD is limited by several problems.

First, not all children benefit sufficiently from treatment. In general, after standardized CBT, average symptom improvement is about 50%, with large individual differences [10,11]. The combination of CBT with pharmacotherapy is an option for partial responders and nonresponders, but recent studies have cast doubts on the additional effect of medication [12,13]. Furthermore, use of medication entails several disadvantages, such as possible adverse effects, a heightened chance of relapse by discontinuation, and unknown effects in the long term [14]. This highlights the need for new ways to improve treatment for pediatric OCD.

Second, there are organizational and practical barriers to treatment. Particularly in remote areas, CBT is not always available, and in many places, there has been a long tradition of a shortage of experienced therapists and long waitlists for treatment [15-18]. Practical problems with scheduling, treatment associated costs, disorder-specific symptoms that restrict mobility, shame, and stigma can further limit accessibility to treatment [16,19-22].

In parallel, the use of digital technology in child mental health care is rapidly increasing [23]. Technologies (computers, internet, mobile devices, and apps) offer a unique opportunity to address several limitations associated with traditional treatment, such as access, suitability, expense, and stigma. In addition, new technologies can be appealing to children and adolescents, which may increase treatment compliance and motivation.

Several types of technology-based CBT (tCBT) programs for OCD have been developed and implemented, including online bibliotherapy, online self-help therapy, therapist-supported computerized CBT, smartphone apps, traditional CBT delivered via telephone or videoconferencing, and combinations of these forms [22,24-27]. Preliminary evidence shows that these programs yield positive effects overall. A meta-analysis on tCBT for OCD, which was based on eight randomized controlled trials (N=420, including youth, n=31), showed a large effect size for tCBT (*d*=1.18, CI 0.80-1.56) [25]. Moreover, tCBT was found to be superior to control conditions (waitlist and active controls, *d*=0.82), and no difference was found in efficacy between tCBT and traditional therapist-delivered CBT [25]. Results from a recent systematic review on tCBT for pediatric OCD (N=96) indicated that tCBT can be a feasible and acceptable treatment for youth with OCD [24].

However, tCBT for OCD is still in its infancy. Current evidence is limited by small numbers of trials, small sample sizes, methodological shortcomings, and focus on adults in most studies. In addition, tCBT programs vary greatly in format, duration, intensity, length, and their specific aims, making it hard to draw firm conclusions. This stresses the need for further research, especially in children with OCD.

In this protocol, we propose an *enhanced* cognitive behavioral therapy (eCBT) package for pediatric OCD, integrating modern internet technology and traditional CBT, in order to improve treatment response as well as user friendliness.

### eCBT for Pediatric OCD

eCBT is an innovative treatment package for children and adolescents with OCD, which has been developed by academic OCD experts, clinicians, information technology and media developers, and service users. eCBT integrates modern technology with well-validated principles of CBT, with the aim to address some challenges faced by traditional therapy. eCBT employs the Norwegian [28] and Dutch [29] treatment manuals for CBT for pediatric OCD. For both protocols, effectiveness has been demonstrated [11,30]. Equivalent to traditional CBT, eCBT contains psychoeducation, exposure with response prevention (ERP), cognitive interventions, and relapse prevention. Parents are actively involved in the treatment. eCBT enhances traditional CBT by offering treatment at home via a webcam in addition to face-to-face sessions, more frequent therapist contact, and an app system to support and monitor treatment. Taking into account the shortage of experienced therapists and high societal health care costs, total therapist time for eCBT is kept equivalent to traditional CBT.

#### Treatment Components

The following five closely linked components are integrated in the eCBT treatment process: videoconferencing sessions in combination with face-to-face sessions, an app system, a psychoeducation tool, and frequent online ratings with direct feedback to the patient. We describe these components in more detail below.

eCBT combines face-to-face treatment sessions with videoconferencing sessions from home. During the videoconferencing sessions, therapists assist children in ERP exercises at home or at other places that elicit OCD symptoms. The videoconferencing sessions aim to improve the ecological validity of the treatment and to encourage generalization of CBT principles by extending treatment from the therapist’s office to settings in which the problems naturally occur. In addition, treatment at home may be more convenient and may reduce travelling time, costs, and stigma. Children and therapists have access to the video-teleconferencing software via their smartphones, using Cisco Webex Teams [31]. Face-to-face sessions, allowing for full contact, may facilitate the building of therapeutic alliance and may provide the therapist with other information since observations are not limited to the scope of the webcam.

The app system consists of a smartphone app for children, a smartphone app for parents, and a web-based application for therapists on the computer, which are all interconnected. The main goals of the app system are to increase motivation and treatment adherence, and to encourage parents’ involvement in the treatment process. The app system further contributes to personalizing treatment to individual needs. The app provides information about OCD and CBT (psychoeducation videos), supports and structures ERP exercises at home, and closely monitors treatment progress. The web-based application for therapists has a coordinating and monitoring function. The app can be used in the treatment sessions together with the therapist and independently at home. Multimedia Appendix 1 provides an overview of the app system.

**Figure 1:**
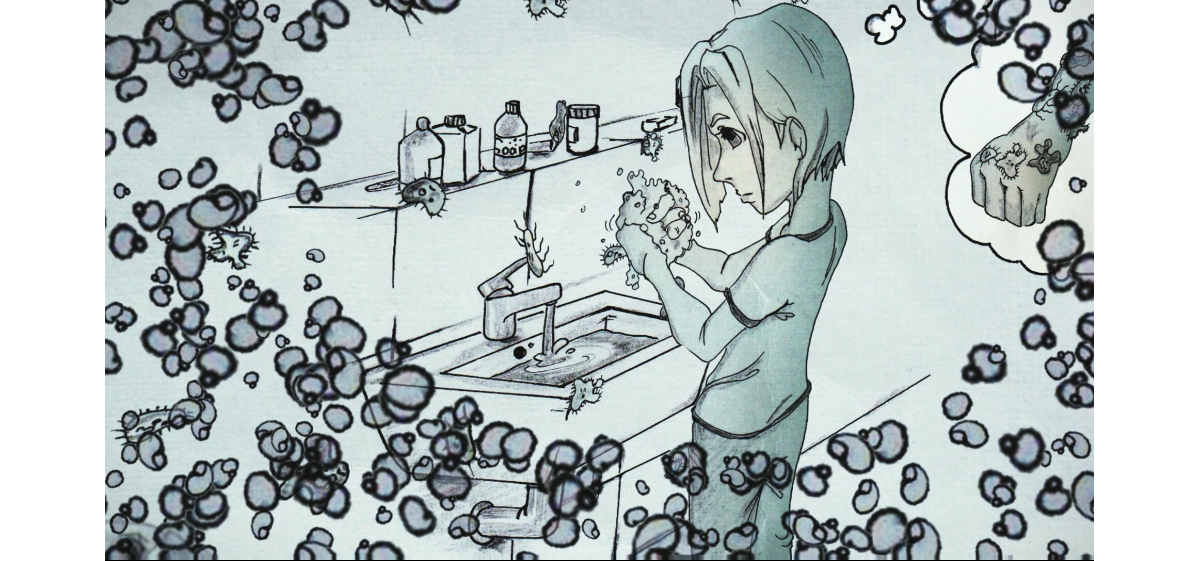
Psychoeducation tool.

The app system is fully integrated in the treatment process, starting in the first session with the psychoeducation tool. The psychoeducation tool contains four video stories showing animated narratives of children with OCD. The aim is to provide information about OCD and treatment (CBT), to increase insight in an attractive and accessible way, to give recognition to a patient’s struggle with OCD and take away shame, and to provide hope and motivation for treatment. The videos, displaying cartoons voiced over by a child or a parent, show how OCD has interfered in the lives of these children and their families, and show their experiences with treatment. The tone is positive and encouraging. The portraits represent children of different ages and both sexes, with different OCD symptomatology to facilitate recognition and identification with one of the portraits. Figure 1 displays a picture of one of the video stories. Multimedia Appendix 2 provides a description of the stories.

The app is also used to facilitate listing and monitoring of OCD symptoms. OCD symptoms can be entered via the therapist application during the treatment sessions or directly in the child and parent apps. Symptoms are scored on a subjective units of distress (SUD) scale. The three symptoms identified by the child and parents as most important are marked, forming the *top problems* measure that is used for idiographic patient-guided assessment of treatment progress [32]. Parents can make a list of the child’s symptoms from their perspective, for example, in case of young children who are not able to list the symptoms themselves and in the case of different views on the symptoms between parents and the child. This approach allows the therapist to get a more complete picture of the OCD and keep parents involved, and it may facilitate discussion when the child and parents disagree, opening the way to a shared vision.

The app further contains a feature to structure and support ERP exercises at home. During the treatment sessions, ERP exercises are described in the app and can be displayed at any moment in the child and parent versions of the app. The child can receive daily reminders for the ERP exercises. When the child activates an ERP exercise in the app, the description of the exercise appears, followed by a button to confirm the start and end of the exercise, and finally, an evaluation question (“How much discomfort did you have during the exercise?”; visual analog scale [VAS]). The app contains a virtual reward system for the number of completed ERP exercises to keep the child motivated. The description of the ERP exercises in the parents’ app keeps parents informed and may facilitate parents to support the exercises. Therapists can add, modify, and monitor exercises via the therapist application.

The child can build a personal support and relapse prevention plan via the app. Any kind of support (ie, coping strategies and tools for dealing with distress) in overcoming OCD can be added to the “toolbox” in the form of text, images, pictures, and video and audio files. The toolbox is a working file that is continuously supplemented and refined throughout the treatment. At the end of the treatment, an individualized relapse prevention plan is added to the toolbox. This plan can be exported to a PDF file, allowing for a paper version of the plan as well.

**Figure 2:**
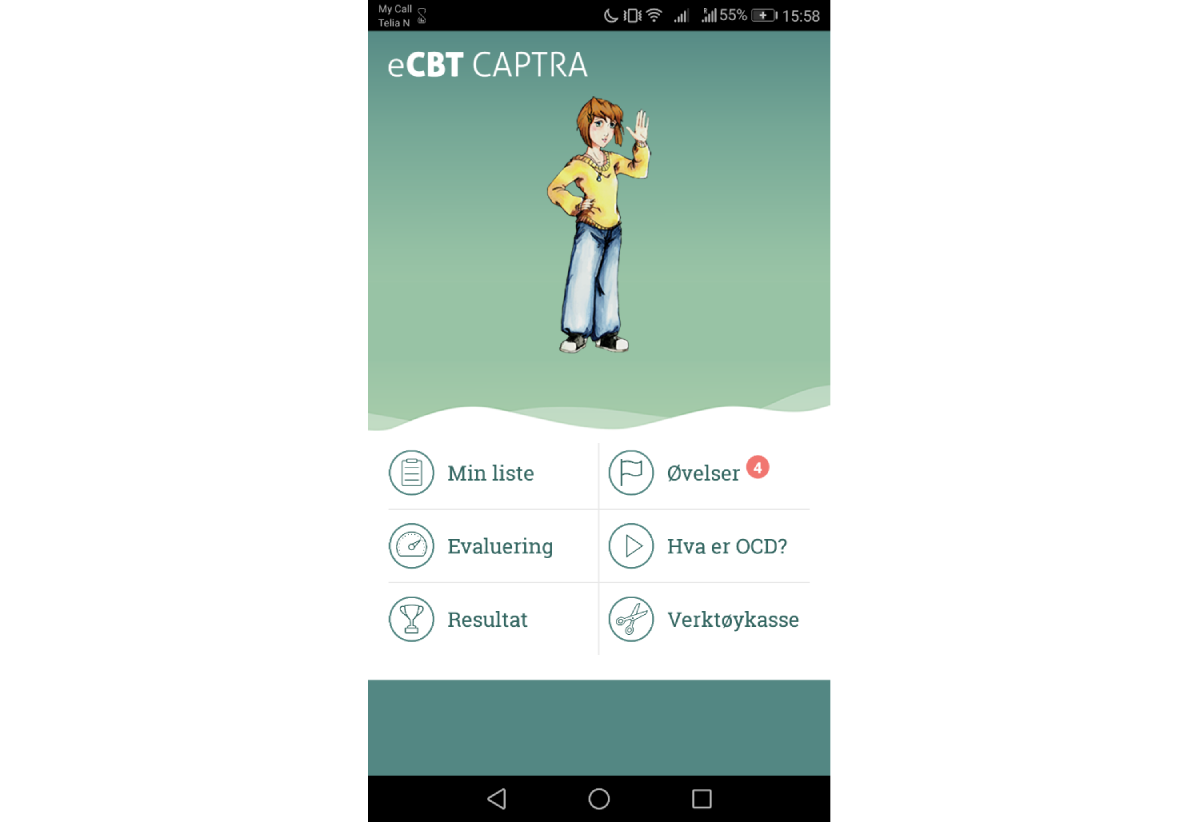
Screenshot of the app for children.

The child and parents are encouraged to daily rate OCD severity and overall psychological well-being using short idiographic ratings in the app with direct feedback to the patient and the therapist (Multimedia Appendix 3). In addition, the three OCD-related problems identified by the child and parents as most important (*top problems* measure) are evaluated weekly in the app. Reminders can be set for completing the ratings. Results (visually displayed in graphs showing progress during the last week and last 6 months) are directly accessible via the child and parent apps, since direct feedback to patients may enhance motivation and thereby treatment effect [33]. In addition, the outcomes provide the therapist with actual information. Signs of noncompliance can be monitored regularly, and early steps can be taken to address problems. Figure 2 displays a screenshot of the app.

#### Treatment Process

eCBT covers a 14-week treatment period. The first part of the treatment (weeks 1-5) consists of weekly face-to-face sessions, equivalent to traditional CBT. Regular face-to-face sessions allow the therapist to start therapy in full contact with the child and the parents in order to build therapeutic alliance and establish treatment principles. However, as soon as the child starts with ERP exercises at home (week 2), an additional videoconferencing meeting is scheduled, resulting in two appointments with the therapist per week. During the videoconferencing sessions, the therapist guides the child while carrying out an ERP exercise at home or at another location if applicable. In this way, the therapist can provide extra support to the child when performing ERP exercises and solve problems directly. In the second part of the treatment, from week 6 onwards, the frequency of the face-to-face sessions is reduced from weekly to once in 2 weeks, since the treatment principles are expected to be established by this time and the main focus becomes continuation of ERP exercises. From this point, two videoconferencing sessions (guided ERP at home) and one face-to-face session are scheduled in a 2-week period. This schedule offers more frequent therapist contact than the usual weekly sessions in traditional CBT and provides the therapist with extra tools to ensure adequate execution of ERP exercises in a natural environment.

In the first face-to-face session (week 1), the therapist provides psychoeducation about OCD and treatment (CBT), augmented with the psychoeducation tool in the app. The eCBT concept is introduced, including an explanation of the app system. The therapist starts with an OCD symptom inventory, which will be completed during the coming weeks. The child as well as parents identify the three most important OCD-related problems (*top problems* measure). In the coming week, the child and/or parents report OCD symptoms in the app. They also start with ratings via the app. The therapist discusses the outcomes of the ratings during the face-to-face sessions. In the next session (week 2), the therapist and child establish a symptom hierarchy, and ERP exercises are set up. The first ERP exercise is practiced together in the session and will be further practiced at home. An appointment is made for a videoconferencing session later that week to guide the ERP exercise at home. In the third face-to-face session (week 3), the therapist evaluates the ratings (app) with the family and discusses the first experiences with practicing ERP at home. A new ERP exercise is selected (or the previous ERP exercise is adapted) to be practiced during the coming week. The new ERP exercise is first practiced during the session and will be further practiced at home. Based on clinical considerations, the therapist may introduce cognitive interventions (eg, challenging dysfunctional thoughts) during this session. Cognitive interventions are not mandatory but can be used to support the ERP exercises and increase motivation. The manual provides for different cognitive interventions, including guidelines for when to apply these interventions, allowing the therapist to customize the treatment to the child’s needs, capacities, and preferences. The face-to-face session will be followed by a videoconferencing session (guided ERP at home) later that week. From this point, the face-to-face sessions have the same structure and include evaluating the ratings and the ERP exercises practiced at home, preparing new ERP exercises, practicing ERP exercises in the session, and introducing optional cognitive interventions. From week 4, the child works on a personal support plan, which is supported by the “toolbox” feature in the app. The “toolbox” will be continuously supplemented and refined during the treatment. At the end of the treatment (weeks 12-14), an individualized relapse prevention plan is added to the toolbox. Multimedia Appendix 4 provides an overview of the treatment.

### Research Protocol

The research protocol (version March 2020) describes an open study to explore the acceptability, feasibility, and effectiveness of eCBT for pediatric OCD.

### Aims and Hypotheses

The aim of the study is to explore whether eCBT is (1) a feasible intervention in terms of treatment drop out; (2) an acceptable intervention; and (3) an effective intervention for children and adolescents with OCD in terms of positive treatment outcomes and showing noninferiority to traditional CBT (Nordic Long-term OCD Treatment Study [NordLOTS]) [11] for the primary outcome measure (Children’s Yale-Brown Obsessive-Compulsive Scale [CY-BOCS]).

We hypothesize that (1) preterm treatment drop out will be equivalent or lower than that found for traditional CBT (≤10%) [11]; (2) eCBT will be positively evaluated by children and their parents; (3) there will be a considerable reduction in OCD symptoms after treatment; and (4) the treatment outcome (CY-BOCS) for eCBT will show noninferiority to traditional CBT (NordLOTS) [11].

## Methods

### Study Design and Sample Size

This study is an open trial using a historical control design to explore the feasibility, acceptability, and effectiveness of eCBT in children and adolescents with OCD. To examine noninferiority of eCBT to traditional CBT, the treatment outcome for eCBT (CY-BOCS) will be compared to data collected in the NordLOTS [11]. The intended sample size is 30 participants.

### Study Setting and Recruitment

Children who visit the outpatient clinic of the Department of Child and Adolescent Psychiatry at St. Olavs University Hospital (Trondheim) or at BUP Klinikk (Aalesund), and meet the study’s eligibility criteria are informed about the study and asked to consider participation. They receive the opportunity to ask questions and have a reasonable amount of time for consideration. Consenting patients are enrolled in the study. During the intake procedure, a qualified professional confirms OCD diagnosis and comorbid disorders using a semistructured interview (Schedule for Affective Disorders and Schizophrenia for School-Age Children Present and Lifetime Version [K-SADS-PL]) [34] if not completed prior to the trial. A standardized questionnaire is used to collect information about demographics and symptom/treatment history.

### Study Procedures

**Figure 3:**
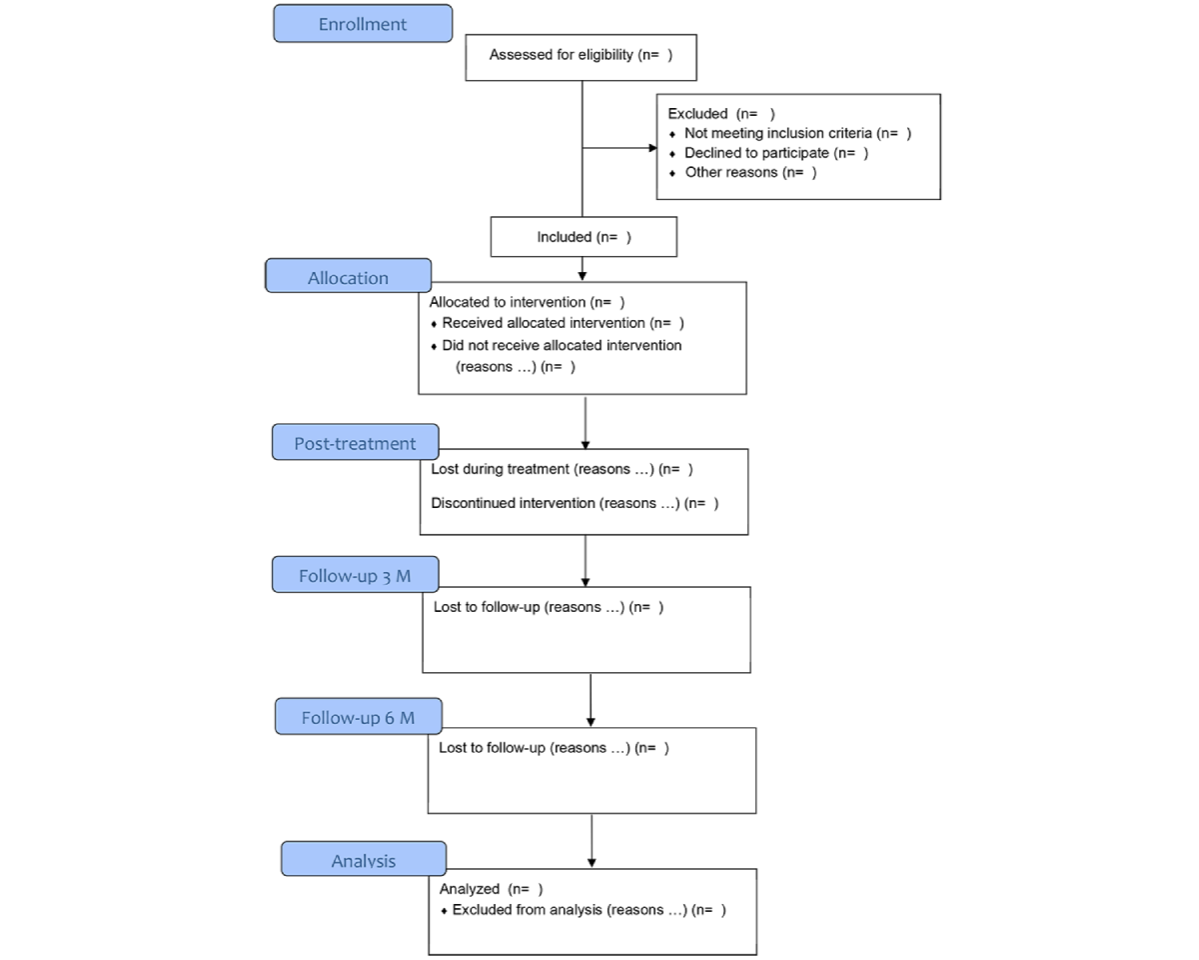
Flow diagram.

All participants receive eCBT. For participants not having a smartphone and for those using an iPhone, an Android smartphone will be lent for the purpose of the study. Concurrent medication is allowed and will be reported during the study. Ongoing psychological treatment for OCD other than eCBT is not allowed. Participants can leave the study at any time for any reason if they wish to do so without any consequences. The investigator can decide to withdraw a participant from the study for urgent medical reasons. The following stop rules for study participation are applied: (1) problems other than OCD requiring acute other treatment (eg, severe depression and suicidal ideation) and (2) severe increase in OCD symptoms and insufficient response to eCBT treatment. Figure 3 shows the flow diagram of the study.

### Participants

To be eligible for study participation, a participant must meet all of the following criteria: (1) age 7-17 years (inclusive); (2) primary Diagnostic and Statistical Manual of Mental Disorders (DSM)-5 diagnosis of OCD; and (3) CY-BOCS score ≥16.

A potential participant meeting any of the following criteria will be excluded from participation: (1) a psychiatric comorbidity that has a higher treatment priority than OCD and makes participation clinically inappropriate (eg, primary anorexia nervosa, depression with suicidality, and psychosis); (2) mental retardation (if suspected based on the level of functioning, or in the presence of neuropsychiatric comorbidity, an IQ test is performed); and (3) insufficient understanding of the Norwegian language.

### Assessments

Assessments are performed pretreatment and posttreatment, and at 3- and 6-month follow-ups. A 12-month follow-up assessment is envisioned.

### Outcome Measures

Outcome measures for treatment acceptability, feasibility, and effectiveness are specified below. Table 1 provides an overview of the assessments. Acceptability involves the following: Client Satisfaction Questionnaire-8 (CSQ-8) [35], primary outcome; a treatment evaluation questionnaire for children and their parents composed of the User Experience Questionnaire (UEQ) [36], and qualitative and quantitative treatment-specific questions; a modified version of the Barriers to Treatment Participation Scale (BTPS) [37], child and parent version (for the aim of this study, we shortened the questionnaire, added a child version, and adapted some items to increase the fit to the eCBT treatment package); and a short qualitative interview examining clinicians’ treatment satisfaction and their suggestions to improve the treatment. Feasibility involves the following: preterm treatment drop out, primary outcome; and number of eligible participants that rejected/accepted eCBT. Effectiveness involves the following: CY-BOCS [38], primary outcome; Child Obsessive-Compulsive Impact Scale-Revised (COIS-R) [39]; and Family Accommodation Scale for OCD–Self-Rated Version (FAS-SR) [40]. Comorbidity/psychological well-being involves the following: Strengths and Difficulties Questionnaire (SDQ) [41]; Child Behavior Checklist (CBCL) [42]; Youth Self-Report Questionnaire (YSR) [42]; Screen for Child Anxiety-Related Emotional Disorders-Revised (SCARED-R) [43,44]; Mood and Feelings Questionnaire (MFQ) [45]; KINDL-R [46]; Children’s Global Assessment Scale (CGAS) [47]; and Clinical Global Impressions Scale-Severity/Improvement (CGI-S, CGI-I) [48]. Other study parameters include the following: study-specific *treatment integrity forms* completed by the therapist following each treatment session and the Trimbos/iMTA Questionnaire for Costs associated with Psychiatric Illness (TiC-P) [49].

**Table 1:** Overview of assessments.

Assessment	Pretreatment	During treatment	Posttreatment	Follow-up 3 months	Follow-up 6 months
CY-BOCS^a^	IE^b^	N/A^c^	IE	IE	IE
CGI-S^d^	IE	N/A	IE	IE	IE
CGI-I^e^	N/A	N/A	IE	IE	IE
CGAS^f^	Therapist	N/A	Therapist	Therapist	Therapist
COIS-R^g^	Child and parent	N/A	Child and parent	Child and parent	Child and parent
FAS^h^	Parent	N/A	Parent	Parent	Parent
CBCL^i^	Parent	N/A	Parent	N/A	Parent
YSR^j^	Child (≥11 years)	N/A	Child (≥11 years)	N/A	Child (≥11 years)
SCARED^k^	Child and parent	N/A	Child and parent	Child and parent	Child and parent
MFQ^l^	Child and parent	N/A	Child and parent	Child and parent	Child and parent
SDQ^m^	Child and parent	N/A	Child and parent	Child and parent	Child and parent
KINDL	Child and parent	N/A	Child and parent	Child and parent	Child and parent
CSQ-8^n^	N/A	N/A	Child and parent	N/A	N/A
Treatment evaluation questionnaire	N/A	N/A	Child and parent	N/A	N/A
BTPS^o^ (modified version)	N/A	N/A	Child and parent	N/A	N/A
TiC-P^p^	Parent	N/A	Parent	Parent	Parent
Session integrity form	N/A	Therapist	N/A	N/A	N/A

^a^CY-BOCS: Children’s Yale-Brown Obsessive-Compulsive Scale.

^b^IE: independent evaluator.

^c^N/A: not applicable.

^d^CGI-S: Clinical Global Impressions Scale-Severity.

^e^CGI-I: Clinical Global Impressions Scale-Improvement.

^f^CGAS: Children’s Global Assessment Scale.

^g^COIS-R: Child Obsessive-Compulsive Impact Scale-Revised.

^h^FAS: Family Accommodation Scale for obsessive-compulsive disorder.

^i^CBCL: Child Behavior Checklist.

^j^YSR: Youth Self-Report Questionnaire.

^k^SCARED: Screen for Child Anxiety-Related Emotional Disorders.

^l^MFQ: Mood and Feelings Questionnaire.

^m^SDQ: Strengths and Difficulties Questionnaire.

^n^CSQ-8: Client Satisfaction Questionnaire-8.

^o^BTPS: Barriers to Treatment Participation Scale.

^p^TiC-P: Trimbos/iMTA Questionnaire for Costs associated with Psychiatric Illness.

### Data Management

All hard-copy forms and informed consent forms will be stored in a secured facility. Protection of participant identity will be guaranteed by assigning study-specific unique participant codes. Only the principle investigator (NS) and the executive investigator (LBE) have access to the key for unique study IDs. Codes will be used to conceal identities in all external communications. Rechecks or later use of the data will be possible using the anonymized data file. Later use of the data will only be possible with consent of the participant. Information (raw data) will be stored for 10 years.

Regular reports are sent to the funding agency. The study is not monitored or audited by an independent party.

### Safety Procedures

Children and parents are encouraged to report the occurrence of adverse events or undesirable treatment effects to their therapists or to the investigator. Therapists report this information in the treatment integrity forms and contact the research team if needed. In addition, a member of the research team (BW) will discuss the occurrence of adverse events and undesirable treatment effects with the therapists at regular times. The investigator will report all serious adverse events that logically could be expected to be related to study participation or eCBT treatment to the sponsor without undue delay after obtaining knowledge of the events.

In case a participant’s condition deteriorates seriously during treatment, the therapist will perform an immediate assessment of the symptoms and will discuss this with the investigator to determine necessary actions.

The additional risk related to study participation for participants is assessed as negligible compared to regular treatment. eCBT follows the treatment principles of CBT, which is the evidence-based treatment for pediatric OCD. In addition, treatment progress and signs of noncompliance are monitored regularly, and immediate steps can be taken when problems are detected. In case of faltering technology (app or webcam), the therapist can be contacted by telephone, email, or face-to-face appointment. Patients can terminate treatment participation at any time and switch to regular treatment if desired. Risks related to technology and security cannot be excluded (ie, hacked data or spyware compromising patient confidentiality). However, security measures are undertaken. Data gathered with the app are stored on a secured server.

### Statistical Analysis

Regarding feasibility and acceptability, descriptive statistics will be provided as follows: CSQ-8, treatment evaluation questionnaire, and modified BTPS for treatment acceptability, and preterm treatment drop out and number of eligible participants that rejected/accepted eCBT for treatment feasibility.

Regarding effectiveness, the treatment effect is expressed as percentage symptom improvement based on the CY-BOCS, the percentage of patients with OCD symptoms in the clinical range (CY-BOCS ≥ 16) and in remission (CY-BOCS ≤ 12), and the percentage of treatment responders (≥35% symptom reduction on the CY-BOCS plus CGI-I rating of 1 or 2 “[very] much improved”) [50]. Effect size (*d*) is calculated by the mean difference in the CY-BOCS score before and after eCBT divided by the SD of the difference in the score before and after eCBT. We will run a series of linear mixed models with treatment outcome (CY-BOCS, CGAS, COIS-R, FAS-SR, CBCL/YSR, SCARED, and MFQ) as the dependent factor and time as the independent factor. An independent *t* test will be performed to compare the treatment outcome (difference in the CY-BOCS score before and after treatment) in this study with the treatment outcome reported for the NordLOTS [11]. 

In terms of other study parameters, for treatment adherence, treatment integrity forms are evaluated by two raters independently, and Cohen kappa will be calculated. To get an impression of treatment costs, outcomes for the TiC-P (related to the SDQ) will be described.

In case of missing assessments, all possible attempts will be made to contact participants.

For feasibility and acceptability analyses, missing data will be described. Acceptability analyses will be conducted on cases having complete data on these measures after treatment.

Regarding treatment outcome, an algorithm for handling missing data is integrated in linear mixed model analyses. Cases with missing data at baseline will be excluded from analyses.

### Sample Size

As the intervention concerns an innovative treatment, the study is primarily aimed at studying acceptability and feasibility. For this goal, a power calculation cannot be performed.

To explore noninferiority of eCBT to traditional CBT (as delivered in the NordLOTS), we will use a historical control design. A power calculation (noninferiority margin set at 4 points on the CY-BOCS) shows that 21 participants per treatment arm would be sufficient to show noninferiority (80% power).

## Results

The study has been approved by the Regionale komiteer for medisinsk og helsefaglig forskningsetikk (REK 2016/716/REK nord) and has been registered in the ISRCTN registry (ID: ISRCTN37530113). The study will be conducted according to the principles of the Declaration of Helsinki (version October 19, 2013; WMA, 2013) [51] and in accordance with the Medical Research Involving Human Subjects Act (WMO) and Good Clinical Practice (GCP) standards.

Multimedia Appendix 5 provides a summary of the trial registration data. Informed consent will be obtained prior to enrollment in the study. Inclusion started on September 04, 2017. Data collection is ongoing. The results will be published in peer-reviewed academic journals, presented at scientific conferences, and communicated to the participants and patient organizations. International Committee of Medical Journal Editors (ICMJE) criteria on contributorship and authorship are applied.

## Discussion

This study is the first step in testing the acceptability, feasibility, and preliminary effectiveness of eCBT. In case of positive results, future steps include improving the eCBT treatment package based on feedback from service users, examining cost-effectiveness in a randomized controlled trial, and making the package available to clinicians and other service providers treating OCD in children and adolescents.

Although eCBT has not been developed with the intention to overcome all barriers to treatment, we aim to improve treatment response by offering a more focused application of CBT principles in a user-friendly way. A future step would be to examine which approach works best for which patients.

## Appendix

Multimedia Appendix 1 Overview of the app system.

Multimedia Appendix 2 Psychoeducation tool: stories.

Multimedia Appendix 3 Questions for monitoring treatment progress via the app system.

Multimedia Appendix 4 Enhanced cognitive behavioral therapy: treatment overview.

Multimedia Appendix 5 Summary of trial registration data.

## Abbreviations

BTPS: Barriers to Treatment Participation Scale
CBCL: Child Behavior Checklist
CBT: cognitive behavioral therapy
CGAS: Children’s Global Assessment Scale
CGI-I: Clinical Global Impressions Scale-Improvement
CGI-S: Clinical Global Impressions Scale-Severity
COIS-R: Child Obsessive-Compulsive Impact Scale-Revised
CSQ-8: Client Satisfaction Questionnaire-8
CY-BOCS: Children’s Yale-Brown Obsessive-Compulsive Scale
eCBT: enhanced cognitive behavioral therapy
ERP: exposure with response prevention
FAS-SR: Family Accommodation Scale for OCD–Self-Rated Version
K-SADS-PL: Schedule for Affective Disorders and Schizophrenia for School-Age Children Present and Lifetime Version
MFQ: Mood and Feelings Questionnaire
OCD: obsessive-compulsive disorder
SCARED-R: Screen for Child Anxiety-Related Emotional Disorders-Revised
SDQ: Strengths and Difficulties Questionnaire
tCBT: technology-based cognitive behavioral therapy
TiC-P: Trimbos/iMTA Questionnaire for Costs associated with Psychiatric Illness
UEQ: User Experience Questionnaire
YSR: Youth Self-Report Questionnaire
