# Supplementary material for: Feasibility, Acceptability, and Effectiveness of Enhanced Cognitive Behavioral Therapy (eCBT) for Children and Adolescents With Obsessive-Compulsive Disorder: Protocol for an Open Trial and Therapeutic Intervention
Source: JMIR Res Protoc. 2020 Dec 18;9(12):e24057. doi: 10.2196/24057 (PMC7775822; doi:10.2196/24057)
Supplement: Multimedia Appendix 1 [file resprot_v9i12e24057_app1.pdf]

App for children	App for parents	Application for therapists
Psychoeducation tool (video files)	Psychoeducation tool (video files)	
List of OCD (to list symptoms, add SUD scores, 3 Top Problems)	List of OCD (to view the child's list of OCD symptoms[1]; list OCD symptoms (optional); 3 top problems)	List of OCD (to view the child's and parents' list of symptoms; add symptoms and SUD scores, mark 3 Top Problems)
ERP-exercises (to view ERP-exercises, administrate and evaluate exercises; reward system)	ERP-exercises (to view ERP-exercises)	ERP-exercises (to manage ERP-exercises)
Ratings and results (daily and weekly ratings, graphs with results)	Ratings and results (daily and weekly ratings, graphs with results[1])	Results (to view the results of the child's and parents' ratings)
Toolbox (to create a personalized support and relapse prevention plan)	Toolbox[1] (to view the child's toolbox)	Toolbox (to monitor and manage the child's toolbox and relapse prevention plan)
Reminders (reminders for ERP-exercises, assessments, treatment appointments)	Reminders (reminders for assessments)	
		Sessions (overview of treatment appointments; to add/edit appointments)
		Settings (to edit usernames and passwords; to set reminders for the child and parents; define data sharing with the parents[1])
		Users (overview of patients; to create new users; (de)activate users)
