# Supplementary material for: Feasibility, Acceptability, and Effectiveness of Enhanced Cognitive Behavioral Therapy (eCBT) for Children and Adolescents With Obsessive-Compulsive Disorder: Protocol for an Open Trial and Therapeutic Intervention
Source: JMIR Res Protoc. 2020 Dec 18;9(12):e24057. doi: 10.2196/24057 (PMC7775822; doi:10.2196/24057)
Supplement: Multimedia Appendix 2 [file resprot_v9i12e24057_app2.pdf]

Tom

This portrait tells the story of a 13-year-old boy, Tom, with symmetry compulsions and 'just right' experiences. He likes playing football, but the OCD rituals (touching lines) caused a lot of problems. OCD also impaired his schoolwork. Tom's teacher talked to his parents. Both parents have a history with OCD, and recognized the symptoms. Tom and his parents decided to consult a therapist. Tom tells about his experiences with CBT, mainly focusing on ERP. He explains that he found out that ERP-exercises can be difficult but helpful. The successes that he achieved, and the support from the therapist, his parents and other adults (teacher, football coach), helped him to keep going. At the end, he enjoys playing football again, and completes schoolwork without compulsions. The story ends with Tom's advice to other children: to commit to ERP and do not give up.

Liv

The story of Liv depicts a 15-year-old girl with contamination fear. Liv describes the onset and increase of her OCD symptoms, including her obsessions, cleaning rituals, and avoidance behavior. OCD led to significant impairment; Liv did not leave the house anymore and she had struggles with her sister and parents. Liv and her parents discovered that the most helpful approach was understanding combined with clear appointments about what was (not) permitted regarding her cleaning rituals. Liv's portrait further provides a picture of the treatment process, integrating psychoeducation about OCD and CBT principles. Liv points out feelings of shame and considerations in the social sphere: do I tell about my OCD?; how will other people react? The story ends positive, with hope for the future.

Jan

The 17-year-old Jan tells about his long-time struggle with OCD, his fears (transformation obsessions, magical thinking, and unacceptable thoughts), the rituals, and avoidance behavior. Jan is worried about becoming crazy, prompted by the irrational character of the symptoms. Due to OCD, he had fights with his parents, was often too late for school, and did not see his friends anymore. He tried to hide his OCD as long as possible, but in the end he told his parents. This led to a turnaround. His parents responded with understanding, and Jan started with CBT. He describes the treatment process, integrating information about ERP and how to deal with obsessions. At the end, Jan explains that although OCD is in remission now, the fears, obsessions and rituals sometimes return. He addresses relapse prevention, and ends with some advice to other children with OCD.

Parents

This portrait tells the story of a mother and her daughter with severe OCD. The mother provides a lively description how OCD affected not only her daughter's life, but all family's life. The story addresses family accommodation, parents' dilemmas, worries and related emotions like frustration, despair, and the feeling of being powerless, and provides information about treatment from a parent's perspective. The parents portrayed in this story tried different approaches – accommodating to OCD versus being strict – without success. The situation worsened and they brought their daughter to a therapist. During treatment, these parents learned to set limits regarding OCD while being supportive for their child. The treatment process went with ups and downs, but overall leading to improvement. The story ends on a positive note, with the girl doing much better and parents having confidence in the future again, and a motivating message to other parents.
