# Supplementary material for: Feasibility, Acceptability, and Effectiveness of Enhanced Cognitive Behavioral Therapy (eCBT) for Children and Adolescents With Obsessive-Compulsive Disorder: Protocol for an Open Trial and Therapeutic Intervention
Source: JMIR Res Protoc. 2020 Dec 18;9(12):e24057. doi: 10.2196/24057 (PMC7775822; doi:10.2196/24057)
Supplement: Multimedia Appendix 3 [file resprot_v9i12e24057_app3.pdf]

Daily questions		Answer scale (VAS 0-10)
**Child**	How much has OCD messed things up for you today?	not at all–very much
	Did you avoid situations today because of your OCD?	not at all–almost all situations
	How would you rate your mood today?	unhappy–happy<br>anxious–relaxed
	Overall, how would you rate your day?	a miserable day–an exceptional day
**Parents**	How much did OCD interfere in your child's activities today?	not at all–very much
	Did your child avoid situations today because of his/her OCD?	none–almost all situations
	To what extent did your child's OCD interfere in family life?	not at all–a significant amount
	To what extent were family members involved in your child's OCD rituals?	not at all–very much
**Weekly questions**		**Answer scale (VAS 0-10)**
**Child**	[Top problem 1] How much was this a problem for you last week?	not at all–very much
	[Top problem 2] How much was this a problem for you last week?	not at all–very much
	[Top problem 3] How much was this a problem for you last week?	not at all–very much
**Parents**	[Top problem 1]<br>How much was this a problem for your child last week?	not at all–very much
	[Top problem 2]<br>How much was this a problem for your child last week?	not at all–very much
	[Top problem 3]<br>How much was this a problem for your child last week?	not at all–very much
