# Supplementary material for: Feasibility, Acceptability, and Effectiveness of Enhanced Cognitive Behavioral Therapy (eCBT) for Children and Adolescents With Obsessive-Compulsive Disorder: Protocol for an Open Trial and Therapeutic Intervention
Source: JMIR Res Protoc. 2020 Dec 18;9(12):e24057. doi: 10.2196/24057 (PMC7775822; doi:10.2196/24057)
Supplement: Multimedia Appendix 4 [file resprot_v9i12e24057_app4.pdf]

Week	Appointment[1]	Therapeutic Intervention[2]	Enhancement
**Week 1**	**Face-to-face** (45 minutes)	Introduction to the eCBT concept<br>Introduction app<br>Psychoeducation<br>OC symptom inventory, 3 Top Problems<br>Preparation homework: administration of symptoms	App system:<br>- psychoeducation tool<br>- listing OC symptoms<br>- assessments and outcomes<br>- reminders
**Week 2**	**Face-to-face** (45 minutes)	Evaluation ratings<br>OC symptom inventory and hierarchy<br>Start ERP: set up ERP-exercises; practicing ERP in the session<br>Prepare homework (ERP)<br>Evaluation experiences with the app and solving problems	App system:<br>- ratings and results<br>- listing OC symptoms<br>- ERP-exercises<br>- reminders
	**Videoconferencing** (15 minutes)	Therapist-guided ERP at home	Videoconferencing software
**Week 3**	**Face-to-face** (45 minutes)	Evaluation ratings<br>Update list OC symptoms<br>Evaluation ERP-exercises (homework)<br>ERP: prepare new ERP-exercise; practicing ERP in the session<br>Optional: Cognitive interventions (CI)<br>Preparation homework: ERP	App system:<br>- ratings and results<br>- listing OC symptoms<br>- ERP-exercises<br>- reminders
	**Videoconferencing** (15 minutes)	Therapist-guided ERP at home	Videoconferencing software
**Week 4**	**Face-to-face** (45 minutes)	Evaluation ratings<br>Update list OC symptoms<br>Evaluation ERP-exercises (homework)<br>ERP: prepare new ERP-exercise; practicing ERP in the session<br>CI (optional)<br>Introduction toolbox<br>Preparation homework: ERP	App system:<br>- ratings and results<br>- listing OC symptoms<br>- ERP-exercises<br>- toolbox<br>- reminders
	**Videoconferencing** (15 minutes)	Therapist-guided ERP at home	Videoconferencing software
**Week 5**	**Face-to-face** (45 minutes)	Evaluation ratings<br>Update list OC symptoms<br>Evaluation ERP-exercises (homework)<br>ERP: prepare new ERP-exercise; practicing ERP in the session<br>CI (optional)<br>Toolbox<br>Preparation homework: ERP	App system:<br>- ratings and results<br>- listing OC symptoms<br>- ERP-exercises<br>- toolbox<br>- reminders
	**Videoconferencing**	Therapist-guided ERP at home	Videoconferencing software

	(15 minutes)		
**Week 6 & 7**	**Face-to-face** (45 minutes)	Evaluation ratings<br>Update list OC symptoms<br>Evaluation ERP-exercises (homework)<br>ERP: prepare new ERP-exercise; practicing ERP in the session<br>CI (optional)<br>Toolbox<br>Preparation homework: ERP	App system:<br>- ratings and results<br>- listing OC symptoms<br>- ERP-exercises<br>- toolbox<br>- reminders
	**Videoconferencing** (15 minutes)	Therapist-guided ERP at home	Videoconferencing software
	**Videoconferencing** (15 minutes)	Therapist-guided ERP at home	Videoconferencing software
**Week 8 & 9**	**Face-to-face** (45 minutes)	Evaluation ratings<br>Update list OC symptoms<br>Evaluation ERP-exercises (homework)<br>ERP: prepare new ERP-exercise; practicing ERP in the session<br>CI (optional)<br>Toolbox<br>Preparation homework: ERP	App system:<br>- ratings and results<br>- listing OC symptoms<br>- ERP-exercises<br>- toolbox<br>- reminders
	**Videoconferencing** (15 minutes)	Therapist-guided ERP at home	Videoconferencing software
	**Videoconferencing** (15 minutes)	Therapist-guided ERP at home	Videoconferencing software
**Week 10 & 11**	**Face-to-face** (45 minutes)	Evaluation ratings<br>Update list OC symptoms<br>Evaluation ERP-exercises (homework)<br>ERP: prepare new ERP-exercise; practicing ERP in the session<br>CI (optional)<br>Toolbox<br>Preparation homework: ERP	App system:<br>- ratings and results<br>- listing OC symptoms<br>- ERP-exercises<br>- toolbox<br>- reminders
	**Videoconferencing** (15 minutes)	Therapist-guided ERP at home	Videoconferencing software
	**Videoconferencing** (15 minutes)	Therapist-guided ERP at home	Videoconferencing software
**Week 12 & 13**	**Face-to-face** (45 minutes)	Evaluation ratings<br>Update list OC symptoms<br>Evaluation ERP-exercises (homework)<br>ERP: prepare new ERP-exercise; practicing ERP in the session<br>CI (optional)<br>Toolbox<br>Relapse prevention plan	App system:<br>- ratings and results<br>- listing OC symptoms<br>- ERP-exercises<br>- toolbox, incl relapse prevention plan<br>- reminders

		Preparation homework: ERP / relapse prevention	
	**Videoconferencing** (15 minutes)	Therapist-guided ERP at home	Videoconferencing software
	**Videoconferencing** (15 minutes)	Therapist-guided ERP at home	Videoconferencing software
**Week 14**	**Face-to-face** (45 minutes)	Evaluation ratings<br>Update list OC symptoms<br>Evaluation ERP-exercises / relapse prevention (homework)<br>Refine relapse prevention plan	App system:<br>- ratings and results<br>- listing OC symptoms<br>- ERP-exercises<br>- toolbox, incl relapse prevention plan

Note: [1]Extra therapist contact via videoconferencing (duration: 15 minutes) is compensated by a reduction of face-to-face sessions (during: 45 minutes) in the second part of the treatment; [2]For clinical considerations (e.g., in case of a comorbid disorder), individual adaptions to the treatment schedule are allowed as long as the therapist adheres to the treatment principles and content as described in the manual.
