# Supplementary material for: Feasibility, Acceptability, and Effectiveness of Enhanced Cognitive Behavioral Therapy (eCBT) for Children and Adolescents With Obsessive-Compulsive Disorder: Protocol for an Open Trial and Therapeutic Intervention
Source: JMIR Res Protoc. 2020 Dec 18;9(12):e24057. doi: 10.2196/24057 (PMC7775822; doi:10.2196/24057)
Supplement: Multimedia Appendix 5 [file resprot_v9i12e24057_app5.pdf]

Multimedia Appendix 5. Summary of trial registration data.

Data category	Information
Primary registry and trial identifying number	ISRCTN37530113
Date of registration in the primary registry	January 31, 2020
Secondary identifying number	N/A[a]
Sources of monetary or material support	Central Norway Regional Health Authority<br>Norges Teknisk-Naturvitenskapelige Universitet
Primary sponsor	Central Norway Regional Health Authority (Samarbeidsorganet mellom Helse Midt-Norge RHF)
Secondary sponsors	N/A
Contact for public queries	NS, BW, and LHW
Contact for scientific queries	NS, BW, and LHW
Public title	N/A
Scientific title	Acceptability and feasibility of enhanced cognitive behavioral therapy (eCBT) for children and adolescents with obsessive compulsive disorder: an open trial
Countries of recruitment	Norway
Health conditions or problems studied	Pediatric OCD[b]
Intervention	Enhanced cognitive behavioral therapy

Key inclusion and exclusion criteria	Inclusion: age 7-17 years (inclusive); a primary DSM 5 diagnosis of OCD; CY-BOCS[c] score ≥16
	Exclusion: A psychiatric comorbidity that has a higher treatment priority than OCD and makes participation clinically inappropriate; estimated IQ below 70; insufficient understanding of the Norwegian or English language
Study type	Open trial; Interventional; Primary purpose: treatment
Date of first enrollment	September 04, 2017
Target sample size	30
Recruitment status	Recruitment completed; data collection ongoing
Primary outcomes	CSQ-8[d]; preterm treatment drop-out; CY-BOCS
Key secondary outcomes	Acceptability: study-specific *treatment evaluation questionnaire* including the UEQ[e]; a modified version of the BTPS[f]<br>Effectiveness: COIS-R[g]; FAS-SR[h]; SDQ[i]; CBCL[j]/YSR[k]; SCARED-R[l]; MFQ[m]; KINDL-R; CGAS[n]; CGI-I/S[o]

[a]N/A: not applicable.
[b]OCD: obsessive-compulsive disorder.
[c]CY-BOCS: Children’s Yale-Brown Obsessive-Compulsive Scale.
[d]CSQ-8: Client Satisfaction Questionnaire-8.
[e]UEQ: User Experience Questionnaire.
[f]BTPS: Barriers to Treatment Participation Scale.
[g]COIS-R: Child Obsessive-Compulsive Impact Scale-Revised.
[h]FAS-SR: Family Accommodation Scale for OCD–Self-Rated Version.
[i]SDQ: Strengths and Difficulties Questionnaire.
[j]CBCL: Child Behavior Checklist.
[k]YSR: Youth Self-Report Questionnaire.
[l]SCARED-R: Screen for Child Anxiety Related Emotional Disorders-Revised.
[m]MFQ: Mood and Feelings Questionnaire
[n]CGAS: Children’s Global Assessment Scale.
[o]CGI-I/S: Clinical Global Impressions Scale-Improvement/Severity.
